# Chronic stress and poor sleeping habits are associated with self-reported IBS and poor psychological well-being in the general population

**DOI:** 10.1186/s13104-021-05688-4

**Published:** 2021-07-22

**Authors:** Jasmin Zejnelagic, Bodil Ohlsson

**Affiliations:** Department of Internal Medicine, Lund University, Skåne University Hospital, Jan Waldenström Street 15, Floor 5, 20502 Malmö, Sweden

**Keywords:** Chronic stress, Gastrointestinal symptoms, Irritable bowel syndrome, Sleeping habits

## Abstract

**Objective:**

The present population-based study aimed to examine the association of chronic stress and sleeping difficulties with self-reported irritable bowel syndrome (IBS), gastrointestinal (GI) symptoms past 2 weeks, and psychological well-being.

**Results:**

The Malmö Offspring Study included subjects from the general population to complete a questionnaire regarding sociodemographic factors, lifestyle factors, and medical health. Experience of chronic stress during the past or past 5 years was reported. Sleeping patterns included sleeping quality, sleeping hours per day, sleeping onset difficulties, and wake-up frequency. The severity of GI symptoms was measured with the visual analog scale for IBS. Associations of stress and sleeping habits with IBS and GI symptoms were calculated by logistic regression and generalized linear model, adjusted for sociodemographic and lifestyle factors. After exclusion of organic GI disorders or missing values, 2648 participants remained. Participants with self-reported IBS (n = 316) and GI symptoms (n = 459) were often women and smokers. After full adjustment, chronic stress past year was associated with GI symptoms (OR: 1.347; 95% CI 1.030–1.762), whereas stress past 5 years (OR: 1.415; 95% CI 1.058–1.892) and sleeping onset difficulties ≥ 3 times weekly (OR: 2.153: 95% CI 1.228–3.774) were associated with IBS. Stress, poor sleeping quality, sleeping onset difficulties, and IBS/GI symptoms were all associated with poor psychological well-being (p < 0.001).

**Supplementary Information:**

The online version contains supplementary material available at 10.1186/s13104-021-05688-4.

## Introduction

Irritable bowel syndrome (IBS) is very common and the prevalence in the population varies between 1.1% and 45.0% [1–3]. Self-reported IBS means that participants report whether they have IBS or not, after a short description of IBS symptoms [1, 4]. Most of the IBS patients are handled at primary healthcare centers not using the strict Rome criteria [1, 5], but only 21% of subjects experiencing IBS symptoms will consult a physician [4]. However, recruitment to studies is performed at tertiary centers [6]. In addition to the Rome questionnaire [5], visual analog scales (VAS) can assess the degree of specific gastrointestinal (GI) symptoms and psychological well-being [7, 8].

An experience of overwhelming stress has been found in 74% of the population [9] and is believed to cause increased visceral hypersensitivity and exacerbation of the IBS symptoms through its activation of the hypothalamic–pituitary–adrenal (HPA) axis [10, 11].

Sleeping disorders are also common and are associated with a higher incidence of depression, morbidity, and mortality [12, 13], and occurs in a pooled prevalence of 37.6% in IBS [14, 15].

The primary aim of the present population-based study [16] was to examine whether chronic stress and sleeping habits were associated with self-reported IBS and GI symptoms in the general population. The secondary aim was to examine the associations of stress, sleeping habits, and IBS/GI symptoms with psychological well-being.

## Main text

### Material and methods

#### Study participants

The Malmö Diet and Cancer Study (MDCS) (n = 28,098) is a population-based study collected between 1991 and 1996. Later, 6103 individuals were re-examined and constitutes the Malmö Diet and Cancer Cardiovascular Cohort (MDC-CC). The Malmö Offspring Study (MOS) started in 2013 and consists of children and grandchildren to participants in the MDC-CC, with an inclusion rate of 47% [16]. From the total of 4225 participants included in June 2020, 1577 were excluded due to presence of organic bowel diseases or not having answered the questions regarding GI symptoms. Thus, 2648 individuals were finally included in the study (Additional file 1: Figure S1).

### Study questionnaire

The MOS included a web-based survey with questions regarding sociodemographic factors, lifestyle habits, and medical health, based on previous questionnaires used in population-based studies. Questions included information about chronic stress during the past year and during the past 5 years, sleeping quality, sleeping hours per day, sleeping onset difficulties, and wake-up frequency [16]. The participants were interpreted as having self-reported IBS related to Rome III criteria [17] if they answered “yes” to the following question: “Have you several times during a month suffered from abdominal pain related to irregular bowel habits which is called IBS?”.

### Visual analog scale for irritable bowel syndrome

The participants were asked: “Have you experienced GI symptoms during the past 2 weeks?”. If they answered “yes” to this question, they were encouraged to complete the validated VAS-IBS regarding GI symptoms (abdominal pain, diarrhea, constipation, bloating and flatulence, vomiting and nausea, intestinal symptom’s influence on daily life, and psychological well-being) during the past 2 weeks on a scale of 0–100 mm [7, 8].

### Data categorization

Chronic stress during the past year or past 5 years was categorized into yes or no. Sleeping quality was categorized into very good, good, average, bad, or very bad. Sleeping hours per night were categorized regarding the number of hours. How often they experienced troubles to fall asleep or wake-up frequencies were split into never or rarely, < once/week, 1–2 times/week, 3–6 times/week, and almost every night.

Age was grouped into 10-years intervals. Body mass index (BMI) was grouped into normal or underweight, overweight, and obesity [18]. Smoking and snuff using were grouped into never, former, or present users. Drinking frequency last year was separated into ≤ 1 time/month, 2–4 times per month, 2–3 times/week, and ≥ 4 times/week. Drinking amount per occasion was defined with standard glasses (12 g alcohol) and divided into 1–2, 3–4, 5–6, 7–9, or ≥ 10 glasses. Physical activity at work last year was separated into light, intermediate, and hard, and during leisure time into sedentary to moderate or training regularly.

### Statistical analysis

IBM SPSS version 26 was used. Data were not normally distributed and presented as median (interquartile ranges), calculated with Mann–Whitney U-test.

Logistic regression was used to examine the association of self-reported IBS and GI symptoms (dependent variables) with sociodemographic status and lifestyle factors (independent variables). Crude odds ratio (OR) and 95% confidence interval (CI) were calculated for each variable and adjusted for significant parameters in the crude calculations.

Logistic regression was then used to determine the crude association between self-reported IBS and GI symptoms and the independent six variables for stress and sleeping habits, and between IBS and the specific VAS-IBS items divided into quartiles (independent variables), adjusted for significant background characteristics. The full model with all variables and confounders were the main results of the current study.

To test for sex interaction (i) regarding stress and sleeping habits, a multiplicative variable (e.g., sex × age groups) was added in the adjusted models. P-value < 0.05 was considered as statistically significant.

Generalized linear model was used to examine the association between specific VAS-IBS items (dependent variables) in the full model with all variables (predictors). Values are presented as β (beta-value) and 95% CI. P-value ≤ 0.01 was considered as statistically significant, due to several calculations.

## Results

### Population characteristics

Of the 2648 participants, 47.5% (n = 1257) were men and 52.5% (n = 1391) were women. Self-reported IBS was found in 11.9% of the participants (n = 316) and GI symptoms during the past 2 weeks were reported in 17.3% of participants (n = 459) (Additional file 1: Table S1). IBS participants reported a high prevalence of GI symptoms, and poor psychological well-being was present in both participants with IBS (Additional file 1: Table S2) and GI symptoms (22 (7–50) mm vs. 15 (5–29) mm, p < 0.001).

Participants with self-reported IBS or GI symptoms were more often women and smokers. IBS participants drank less amounts of alcohol on fewer occasions, whereas participants with GI symptoms were younger and more often studying or unemployed, compared to those without GI symptoms (Additional file 1: Table S1).

### Associations between chronic stress, sleeping habits, and self-reported IBS and GI symptoms

Experience of chronic stress during the past year and the past 5 years were associated with self-reported IBS and GI symptoms (Tables 1 and 2).

**Table 1:** Associations between chronic stress or sleeping habits and self-reported IBS

	No IBS N = 2332 (88.1%)	IBS N = 316 (11.9%)	Crude OR 95% CI	P-value	Adjusted OR 95% CI	P-value
Chronic stress 1 year						
No (reference)	58.6	43.4	1.000		1.000	
Yes	41.0	56.6	1.868 (1.474–2.369)	< 0.001	1.612 (1.252–2.076)	< 0.001
Missing	0.4					
Chronic stress 5 years						
No (reference)	64.1	47.5	1.000		1.000	
Yes	35.2	52.2	2.002 (1.579–2.537)	< 0.001	1.786 (1.387–2.301)	< 0.001
Missing	0.7	0.3				
Sleeping quality						
Very good (reference)	28.1	19.6	1.000		1.000	
Good	35.9	26.9	1.072 (0.760–1.510)	0.693	1.151 (0.799–1.659)	0.450
Average	26.0	36.4	2.005 (1.444–2.783)	< 0.001	1.987 (1.395–2.831)	< 0.001
Bad	8.7	13.9	2.301 (1.516–3.493)	< 0.001	2.390 (1.521–3.756)	< 0.001
Very bad	0.9	3.2	4.802 (2.176–10.597)	< 0.001	4.909 (2.146–11.231)	< 0.001
Missing	0.4	–				
Sleeping onset difficulty						
Never or rarely (reference)	45.4	28.5	1.000		1.000	
< 1 per week	29.2	29.7	1.627 (1.199–2.206)	0.002	1.533 (1.107–2.123)	0.010
1–2 per week	16.3	22.8	2.224 (1.597–3.097)	< 0.001	2.092 (1.463–2.991)	< 0.001
3–6 per week	4.9	9.2	2.993 (1.888–4.746)	< 0.001	2.821 (1.722–4.619)	< 0.001
Almost every night	3.7	9.8	4.241 (2.668–6.743)	< 0.001	4.549 (2.756–7.509)	< 0.001
Missing	0.5	–				
Sleeping hours per day						
≤ 5 (reference)	6.1	9.2	1.000		1.000	
6	22.5	25.9	0.772 (0.486–1.225)	0.272	0.784 (0.478–1.286)	0.336
7	44.4	36.1	0.543 (0.348–0.845)	0.007	0.589 (0.365–0.950)	0.030
8	21.2	21.2	0.667 (0.416–1.072)	0.094	0.664 (0.398–1.110)	0.119
9	4.5	5.7	0.837 (0.442–1.587)	0.586	0.602 (0.280–1.297)	0.195
≥ 10	0.8	1.9	1.557 (.572–4.237)	0.386	1.230 (0.401–3.771)	0.717
Missing	0.4	–				
Wake-up frequency						
Never or rarely (reference)	31.2	22.5	1.000		1.000	
< 1 per week	24.0	20.3	1.172 (0.822–1.673)	0.381	1.221 (0.839–1.776)	0.297
1–2 per week	18.7	19.0	1.409 (0.980–2.027)	0.065	1.406 (0.953–2.075)	0.086
3–6 per week	12.0	20.6	2.386 (1.658–3.432)	< 0.001	2.144 (1.446–3.178)	< 0.001
Almost every night	13.7	17.4	1.765 (1.212–2.571)	0.003	1.599 (1.060–2.412)	0.025
Missing	0.5					

IBS, Irritable Bowel Syndrome. OR, Odds Ratio. CI, Confidence Interval. Logistic regression adjusted for sex, smoking, drinking frequency, and drinking amount. Values are presented as percentages and OR and 95% CI. P-value < 0.05 was considered statistically significant

**Table 2:** Associations between chronic stress or sleeping habits and GI symptoms during the past 2 weeks

	No symptoms N = 2189 (82.7%)	Symptoms N = 459 (17.3%)	Crude OR 95% CI	P-value	Adjusted OR 95% CI	P-value
Chronic stress 1 year						
No (reference)	59.6	43.6	1.000		1.000	
Yes	40.1	56.2	1.918 (1.565–2.351)	< 0.001	1.510 (1.201–1.897)	< 0.001
Missing	0.4	0.2				
Chronic stress 5 years						
No (reference)	64.1	52.5	1.000		1.000	
Yes	35.3	46.4	1.604 (1.308–1.967)	< 0.001	1.435 (1.137–1.812)	0.002
Missing	0.6	1.1				
Sleeping quality						
Very good (reference)	27.9	23.1	1.000		1.000	
Good	35.4	32.0	1.092 (0.832–1.432)	0.525	1.180 (0.874–1.547)	0.279
Average	26.4	31.4	1.439 (1.092–1.895)	0.010	1.349 (1.082–1.941)	0.059
Bad	8.9	11.3	1.545 (1.068–2.235)	0.021	1.231 (1.007–2.243)	0.361
Very bad	1.1	2.0	2.256 (1.016–5.008)	0.046	1.983 (1.040–5.389)	0.170
Missing	0.4	0.2				
Sleeping onset difficulty						
Never or rarely (reference)	44.4	38.6	1.000		1.000	
< 1 per week	29.7	27.0	1.048 (0.816–1.345)	0.715	0.895 (0.676–1.185)	0.439
1–2 per week	16.6	19.6	1.362 (1.028–1.803)	0.031	1.228 (0.893–1.688)	0.207
3–6 per week	5.1	6.8	1.520 (0.990–2.334)	0.056	1.207 (0.731–1.922)	0.462
Almost every night	3.7	7.6	2.344 (1.529–3.592)	< 0.001	1.680 (0.984–2.870)	0.057
Missing	0.5	0.4				
Sleeping hours per day						
≤ 5 (reference)	6.2	7.6	1.000		1.000	
6	22.8	23.5	0.849 (0.555–1.299)	0.450	0.815 (0.522–1.273)	0.369
7	44.5	38.1	0.703 (0.469–1.053)	0.087	0.683 (0.446–1.047)	0.080
8	21.0	22.4	0.878 (0.572–1.348)	0.553	0.826 (0.524–1.300)	0.408
9	4.3	6.5	1.249 (0.718–2.174)	0.431	0.942 (0.505–1.757)	0.850
≥ 10	0.8	1.5	1.522 (0.589–3.931)	0.385	0.992 (0.332–2.963)	0.988
Missing	0.4	0.2				
Wake-up frequency						
Never or rarely (reference)	31.2	25.1	1.000		1.000	
< 1 per week	23.4	24.0	1.273 (0.957–1.694)	0.097	1.381 (1.027–1.871)	0.046
1–2 per week	18.5	19.8	1.334 (0.987–1.804)	0.060	1.352 (0.964–1.835)	0.081
3–6 per week	12.3	16.1	1.628 (1.177–2.251)	0.003	1.679 (1.144–2.279)	0.006
Almost every night	14.1	14.4	1.273 (0.914–1.772)	0.154	1.060 (0.814–1.681)	0.781
Missing	0.5	0.7				

GI, Gastrointestinal. OR, Odds Ratio. CI, Confidence Interval. Logistic regression adjusted for age, sex, occupation, and smoking. Values are presented as percentages and OR and 95% CI. P-values < 0.05 was considered statistically significant

Poor sleeping quality and sleeping onset difficulties were associated with self-reported IBS, whereas a sleeping duration of 7 h was inversely associated with self-reported IBS. More than three wake-up per week were associated with IBS, with a lower association when wake-up was occurring almost every night (Table 1).

Average sleeping quality and sleeping onset difficulties almost every night tended to be associated with GI symptoms, whereas sleeping duration of 7 h tended to be inversely associated with GI symptoms. A wake-up frequency of 3–6 times per week showed a significant association with GI symptoms (Table 2).

In the full model, chronic stress the past year was associated with GI symptoms, whereas chronic stress the past 5 years and sleeping onset difficulties ≥ 3 times weekly was associated with self-reported IBS (Table 3), without any sex interactions (data not shown).

**Table 3:** Associations between stress and sleeping habits and IBS or GI symptoms in a full model

	IBS		GI symptoms the past 2 weeks	
	Adjusted OR 95% CI	P-value	Adjusted OR 95% CI	P-value
Chronic stress 1 year				
No (reference)	1.000		1.000	
Yes	1.137 (0.848–1.524)	0.392	1.347 (1.030–1.762)	0.030
Chronic stress 5 years				
No (reference)	1.000		1.000	
Yes	1.415 (1.058–1.892)	0.019	1.223 (0.936–1.598)	0.140
Sleeping quality				
Very good (reference)	1.000		1.000	
Good	0.965 (0.644–1.445)	0.862	1.116 (0.800–1.557)	0.518
Average	1.342 (0.842–2.141)	0.216	1.129 (0.751–1.697)	0.560
Bad	1.184 (0.613–2.288)	0.614	0.889 (0.472–1.676)	0.716
Very bad	2.590 (0.928–7.224)	0.069	1.503 (0.475–4.758)	0.488
Sleeping onset difficulty				
Never or rarely (reference)	1.000		1.000	
< 1 per week	1.384 (0.977–1.962)	0.067	0.779 (0.576–1.053)	0.104
1–2 per week	1.673 (1.113–2.514)	0.013	1.036 (0.719–1.495)	0.848
3–6 per week	2.153 (1.228–3.774)	0.007	1.025 (0.584–1.799)	0.932
Almost every night	3.036 (1.630–5.653)	< 0.001	1.474 (0.767–2.833)	0.244
Sleeping hours per day				
≤ 5 (reference)	1.000		1.000	
6	1.137 (0.657–1.966)	0.647	1.007 (0.584–1.738)	0.979
7	1.134 (0.646–1.993)	0.661	0.893 (0.514–1.552)	0.688
8	1.348 (0.742–2.446)	0.327	1.126 (0.627–2.021)	0.691
9	1.211 (0.528–2.778)	0.651	1.809 (0.872–3.755)	0.112
≥ 10	1.712 (0.516–5.679)	0.380	0.840 (0.204–3.462)	0.810
Wake-up frequency				
Never or rarely (reference)	1.000		1.000	
< 1 per week	1.020 (0.687–1.514)	0.923	1.357 (0.971–1.896)	0.074
1–2 per week	0.991 (0.640–1.534)	0.968	1.194 (0.814–1.751)	0.364
3–6 per week	1.331 (0.843–2.102)	0.220	1.489 (0.971–2.284)	0.068
Almost every night	0.877 (0.528–1.456)	0.612	0.872 (0.539–1.411)	0.577

GI, Gastrointestinal. IBS, irritable bowel syndrome. OR, Odds Ratio. CI, Confidence Interval. Logistic regression with all variables for stress and sleeping habits in a full model, adjusted for sex, smoking, drinking frequency, and drinking amount in IBS and age, sex, occupation, and smoking in GI symptoms. Values are presented as OR and 95% CI. P-values < 0.05 was considered statistically significant

### Associations between stress, sleeping habits, and degree of specific GI symptoms and psychological well-being

The only significant findings for severity of specific GI symptoms were that sleeping onset difficulties was associated with constipation and bloating and flatulence, whereas wake-up episodes almost every night was associated with vomiting and nausea. Chronic stress, less sleeping quality, and sleeping onset difficulties were associated with poorer psychological well-being (Additional file 1: Table S3).

## Discussion

The main findings of the present study were: (1) GI symptoms the past 2 weeks were strongly associated with self-reported IBS, but the two cohorts differed in their associations; (2) chronic stress was associated with IBS/GI symptoms; (3) sleeping onset difficulties were associated with self-reported IBS; and (4) stress, sleeping disturbances, and self-reported IBS/GI symptoms were associated with poor psychological well-being.

Lifetime stress may induce mental and physical health problems [19], including IBS [10, 20]. The prevalence of depression and anxiety in IBS in excess to healthy controls was 33% and 19%, respectively [21]; a factor which interacts with the association between stress and IBS [20]. The present study showed that stress is important in IBS, but the degree of specific GI symptoms or their influence on daily life was not affected in contrast to another study [20].

The duration of symptoms may explain the association between chronic stress for 5 years and IBS and chronic stress past year and GI symptoms. GI symptoms may involve participants with both chronic and newly debuted symptoms, representing drug effects or other comorbidity, whereas IBS patients have a fluctuating symptom pattern and may have had a better period the past 2 weeks. A long-lasting GI disorder may however be a great stress factor leading to hypersensitivity [11].

Our findings of an association between shorter sleeping duration and IBS are supported by one study [23], in contrast to another [22]. The association between sleeping onset difficulties and IBS remained in the full model, in alignment with other studies which describe this phenomenon as a dominant symptom [14, 23–25]. Interestingly, most of the IBS patients related their sleeping disturbances to non-IBS symptoms, such as nocturia, back pain, headache, and arthralgias [22], which complicates the research field further. The present study could not confirm a previous association between the degree of abdominal pain or other pain intensity and sleeping disturbances [22, 26, 27]. Instead, sleeping onset difficulties was associated with the degree of constipation, bloating and flatulence, the most prominent symptoms of the general population [4].

Most studies have a cross-sectional design and causality cannot be analyzed [15]. Poor self-reported sleeping quality predicted more abdominal or other pain, anxiety, and fatigue the next day [27, 28]. Similarly, waking episodes predicted abdominal pain and GI distress, but not bowel patterns [22]. Instead of pain, pre-sleep cognitive arousal was the most important predictor of poor sleeping quality [28]. Higher arousal and awakening index were also found in IBS patients and could be related to sleeping fragmentation [24]. Taken together, studies show that low quality of life, depression, and arousal, all affect the quality and efficiency of sleeping [22, 26, 28], at the same time as they are closely related to the experience of pain and IBS symptoms [10, 21, 22, 26–28].

The abovementioned associations make the causality and pathophysiology extremely complex to study and evaluate. Sleeping deprivation has been shown to lead to enhancements of nociception and a diminished capacity to regulate emotional reactivity leading to depression and/or anxiety [29], and it affects the autonomous nervous system and HPA axis in similarity to chronic stress, leading to visceral hypersensitivity [11, 30]. Furthermore, biomarkers for oxidative stress in tears correlated with GI symptoms in IBS patients with sleeping disorders [25].

The strength of the present study is the big number of 2648 unselected subjects included from the general population who seldom actively apply for medical care [4], instead of selected patients from tertiary centers [6]. Furthermore, the stress and sleeping habits variables were adjusted for age, sex, occupation, smoking, and alcohol habits, unlike previous studies form the general population [12, 27].

## Conclusion

In conclusion, subjects with self-reported IBS and GI symptoms in the general population exhibit a strong association with chronic stress. Sleeping onset difficulties are associated with self-reported IBS. Chronic stress, sleeping disturbances, and IBS/GI symptoms are all associated with poor psychological well-being.

## Limitations

No objective assessments of sleeping quality were performed and the open question character in the present study gave room for self-interpretations, in contrast to the validated Pittsburgh Sleep Quality Index (PSQI) or health-related quality of life questionnaires [12, 22, 23, 25, 27]. However, the VAS-IBS is validated for GI symptoms and psychological well-being [7, 8]. Several statistical calculations were performed, but the full model including all variables was considered the main result.

The participants self-reported IBS and the diagnosis was not validated against the Rome questionnaire [5]. The number of questionnaires must be limited when performing large population-based studies aimed to study several health and disease conditions [16]. Nevertheless, previous studies have shown that most of the society is aware of the IBS symptoms and diagnosis [4]. The self-reported IBS prevalence in our cohort was like the 5.5% fulfilling the Rome IV criteria in the Belgian population [1, 4]. Another limitation is the referral to Rome III, since the MOS started in 2013, and the Rome IV criteria were introduced in 2016. No re-examination against Rome IV has been performed.

## Supplementary Information

**Additional file 1: Figure S1.** Study participants. **Table S1.** General population characteristics. **Table S2.** Associations between VAS-IBS and self-reported IBS. **Table S3.** Associations between stress and sleeping habits and specific GI symptoms or psychological well-being.

## Data Availability

The datasets used and/or analyzed during the current study are available from the corresponding author on reasonable request.

## Abbreviations

BMI: Body mass index
CI: Confidence interval
GI: Gastrointestinal
HPA: Hypothalamic-pituitary-adrenal
IBS: Irritable bowel syndrome
MDC-CC: Malmö Diet and Cancer Cardiovascular Cohort
MOS: Malmö Offspring Study
OR: Odds ratio
VAS-IBS: Visual analog scale for irritable bowel syndrome
