# Supplementary material for: Chronic stress and poor sleeping habits are associated with self-reported IBS and poor psychological well-being in the general population
Source: BMC Res Notes. 2021 Jul 22;14:280. doi: 10.1186/s13104-021-05688-4 (PMC8296624; doi:10.1186/s13104-021-05688-4)
Supplement: Supplementary file 1 — Additional file 1: Figure S1. Study participants. Table S1. General population characteristics. Table S2. Associations between VAS-IBS and self-reported IBS. Table S3. Associations between stress and sleeping habits and specific GI symptoms or psychological well-being. [file 13104_2021_5688_MOESM1_ESM.docx]

**Figure S1:** Study participants

Malmö Diet and Cancer Study 1991-1996

N = 28,098

Malmö Diet and Cancer Study – Cardiovascular Cohort 1991-1994

N = 6103

Malmö Offspring Study 2013-2020 June

N = 4225

Organic GI diseases

N = 873

Not answered if IBS

N = 664

Not answered if GI symptoms during the past 2 weeks N = 40

Included in the present study

N = 2648

## **Legend to Figure**

**Figure S1.** A total of 28,098 participants from the general population completed all tests in the Malmö Diet and Cancer Study between 1991 and 1996. The cardiovascular cohort was extracted containing 6103 randomly selected participants. From this subcategory, children and grandchildren were invited and constituted the Malmö Offspring Study (MOS). This study cohort included those from MOS who had answered the following two questions: “Do you several times a month suffer from abdominal pain and irregular bowel habits known as irritable bowel syndrome (IBS)?” and “Have you experienced gastrointestinal (GI) symptoms during the past 2 weeks?”. Participants with any organic GI diseases (celiac disease, Crohn’s disease, ulcerative colitis, lactose intolerance, reflux, and ulcer) were excluded.

**Table S1**. General population characteristics

| **Variables** | **IBS n = 2648** | |  | **GI symptoms past 2 weeks n = 2648** | |  |
| --- | --- | --- | --- | --- | --- | --- |
|  | **No = 88.1%** | **Yes =**  **11.9%** | **OR (95% CI)** | **No = 82.7%** | **Yes = 17.3%** | **OR (95% CI)** |
| **Age** (years) |  |  |  |  |  |  |
| < 30 | 26.3 | 26.3 | 1.000 | 25.4 | 30.9 | 1.000 |
| 30–39 | 15.2 | 19.0 | 1.343 (0.901-1.999) | 15.1 | 18.3 | 1.105 (0.757-1.612) |
| 40–49 | 18.6 | 23.7 | 1.365 (0.925-2.013) | 18.4 | 22.9 | 1.099 (0.756-1.596) |
| 50–59 | 29.0 | 19.9 | 0.730 (0.489-1.090) | 29.4 | 21.1 | 0.603 (0.412-0.883) |
| ≥ 60 | 10.9 | 11.1 | 0.920 (0.556-1.523) | 11.8 | 6.8 | 0.384(0.202-0.733) |
| **Sex** |  |  |  |  |  |  |
| Men | 49.9 | 29.7 | 1.000 | 50.2 | 34.4 | 1.000 |
| Women | 50.1 | 70.3 | 2.156 (1.620-2.868) | 49.8 | 65.6 | 1.770 (1.387-2.259) |
| **BMI** (kg/m^2^) |  |  |  |  |  |  |
| < 25 | 48.0 | 54.7 |  | 47.6 | 54.5 | 1.000 |
| 25.0 – 29.9 | 34.6 | 31.0 |  | 35.3 | 28.8 | 0.899 (0.688-1.176) |
| > 30 | 17.4 | 14.2 |  | 17.0 | 16.8 | 1.025 (0.726-1.446) |
| **Education** |  |  |  |  |  |  |
| Primary school | 6.0 | 6.3 |  | 6.4 | 4.4 | 1.000 |
| Secondary school | 54.3 | 51.9 |  | 54.6 | 51.2 | 1.483 (0.799-2.750) |
| Higher education | 39.3 | 40.5 |  | 38.6 | 43.6 | 1.774 (0.942-3.340) |
| Missing | 0.3 | 1.3 |  | 0.4 | 0.9 |  |
| **Occupation** |  |  |  |  |  |  |
| Working | 69.8 | 64.6 |  | 70.2 | 64.3 | 1.000 |
| Studying | 6.6 | 6.6 |  | 6.0 | 9.2 | 1.597 (1.031-2.473) |
| Sick leave | 0.9 | 1.9 |  | 0.9 | 1.3 | 1.473 (0.565-3.839) |
| Unemployed | 2.4 | 3.2 |  | 2.1 | 4.1 | 2.143 (1.207-3.804) |
| Retired | 2.3 | 1.6 |  | 2.5 | 0.9 | 0.901 (0.281-2.892) |
| Other | 2.0 | 2.5 |  | 2.1 | 2.0 | 0.960 (0.457-2.020) |
| Missing | 16.2 | 19.6 |  | 16.2 | 18.3 |  |
| **Marital status** |  |  |  |  |  |  |
| Living alone | 25.0 | 27.2 |  | 24.7 | 27.9 |  |
| Living together | 66.6 | 63.3 |  | 67.2 | 61.7 |  |
| Other | 8.1 | 9.5 |  | 7.9 | 10.2 |  |
| Missing | 0.2 | 0 |  | 0.2 | 0.2 |  |
| **Smoking** |  |  |  |  |  |  |
| Never | 62.2 | 51.6 | 1.000 | 62.2 | 54.9 | 1.000 |
| Former | 24.0 | 29.1 | 1.744 (1.285-2.367) | 24.0 | 27.2 | 1.518 (1.148-2.007) |
| Present | 13.7 | 19.3 | 1.925 (1.352-2.742) | 13.6 | 17.9 | 1.479 (1.055-2.072) |
| Missing | 0.2 | 0 |  | 0.2 | 0 |  |
| **Snuff using** |  |  |  |  |  |  |
| Never | 79.2 | 79.1 |  | 78.8 | 81.3 |  |
| Former | 8.3 | 9.2 |  | 8.6 | 7.6 |  |
| Present | 11.3 | 10.4 |  | 11.6 | 9.4 |  |
| Missing | 1.1 | 1.3 |  | 1.0 | 1.7 |  |
| **Drinking frequency** |  |  |  |  |  |  |
| Never | 6.0 | 8.9 | 1.000 | 6.1 | 7.4 | 1.000 |
| ≤ 1 time/month | 20.3 | 22.5 | 1.059 (0.772-1.452) | 20.5 | 20.9 | 0.756 (0.450-1.270) |
| 2–4 times/month | 41.8 | 44.3 | 0.841 (0.581-1.218) | 42.2 | 41.6 | 0.803 (0.494-1.306) |
| 2–3 times/week | 27.7 | 22.8 | 0.352 (0.123-1.006) | 27.1 | 27.2 | 0.950 (0.568-1.587) |
| ≥ 4 times/week | 3.9 | 1.3 | - | 3.8 | 2.2 | 0.571 (0.235-1.384) |
| Missing | 0.3 | 0.3 |  | 0.3 | 0.7 |  |
| **Drinking glasses/occasion** |  |  |  |  |  |  |
| 1–2 | 51.0 | 55.4 | 1.000 | 50.9 | 54.2 |  |
| 3–4 | 28.3 | 21.2 | 0.716 (0.522-0.982) | 28.1 | 24.2 |  |
| 5–6 | 9.5 | 9.8 | 1.036 (0.659-1.628) | 9.5 | 9.6 |  |
| 7–9 | 3.9 | 3.2 | 0.929 (0.448-1.927) | 3.9 | 3.3 |  |
| ≥ 10 | 1.0 | 1.3 | 1.661 (0.535-5.162) | 1.1 | 0.9 |  |
| Missing | 6.4 | 9.2 |  | 6.5 | 7.8 |  |
| **Physical activity work** |  |  |  |  |  |  |
| Light | 57.8 | 56.3 |  | 57.0 | 60.8 |  |
| Intermediate | 24.9 | 24.4 |  | 25.4 | 22.4 |  |
| Hard | 13.9 | 13.3 |  | 14.1 | 12.2 |  |
| Missing | 3.4 | 6.0 |  | 3.5 | 4.6 |  |
| **Physical activity leisure** |  |  |  |  |  |  |
| Sedentary to moderate | 16.0 | 19.0 |  | 16.2 | 17.2 |  |
| Training regularly | 37.6 | 38.3 |  | 37.1 | 40.3 |  |
| Missing | 46.4 | 42.7 |  | 46.7 | 42.5 |  |

IBS = Irritable bowel syndrome. BMI = body mass index. OR = odds ratio. CI = confidence interval. Physical activity at work last year was separated into light (standing, driving, or office work), intermediate (walking, cleaning, or laundry), and hard (timberwork, heavier service, or gardening). Physical activity during leisure time was grouped into sedentary to moderate (walking or biking without sweating), or training regularly (exercise > 30 min twice a week). Categorical variables are shown as percentages. Logistic regression included all variables with statistical significance in the crude model and are presented as OR and 95% CI. P < 0.05 was considered statistically significant.

**Table S2**. Associations between VAS-IBS and self-reported IBS

| **VAS-IBS**  **(mm)** | **No IBS = 332** | **IBS = 316** | **Crude OR**  **95% CI** | **P-**  **value** | **Adjusted OR**  **95% CI** | **P-value** |
| --- | --- | --- | --- | --- | --- | --- |
| **Abdominal pain** | 10 (1-27) | 40 (14-60) |  |  |  |  |
| 0-3 |  |  | 1.000 |  | 1.000 |  |
| 4-20 |  |  | 2.250 (1.225-4.134) | 0.009 | 2.451 (1.291-4.656) | 0.006 |
| 21-50 |  |  | 6.659 (3.585-12.369) | <0.001 | 6.269 (3.222-12.201) | <0.001 |
| 51-100 |  |  | 9.545 (4.985-18.278) | <0.001 | 8.611 (4.233-17.516) | <0.001 |
| **Diarrhea** | 10 (1-39) | 31 (8-62) |  |  |  |  |
| 0-2 |  |  | 1.000 |  | 1.000 |  |
| 3-19 |  |  | 1.325 (0.749-2.344) | 0.334 | 1.438 (0.77-2.680) | 0.253 |
| 20-51 |  |  | 2.344 (1.348-4.075) | 0.003 | 2.811 (1.506-5.244) | 0.001 |
| 52-100 |  |  | 4.102 (2.325-7.235) | <0.001 | 4.766 (2.510-9.051) | <0.001 |
| **Constipation** | 4 (0-23) | 32 (4-60) |  |  |  |  |
| 0-0 |  |  | 1.000 |  | 1.000 |  |
| 1-9 |  |  | 0.900 (0.498-1.625) | 0.727 | 0.792 (0.419-1.495) | 0.471 |
| 10-49 |  |  | 1.978 (1.112-3.520) | 0.020 | 2.019 (1.076-3.788) | 0.029 |
| 50-100 |  |  | 5.630 (3.138-10.102) | <0.001 | 3.754 (1.990-7.080) | <0.001 |
| **Bloating and flatulence** | 20 (4-50) | 60 (37-73) |  |  |  |  |
| 0-13 |  |  | 1.000 |  | 1.000 |  |
| 14-40 |  |  | 2.545 (1.360-4.765) | 0.003 | 2.648 (1.363-5.146) | 0.004 |
| 41-67 |  |  | 9.055 (4.844-16.928) | <0.001 | 8.426 (4.289-16.552) | <0.001 |
| 68-100 |  |  | 10.333 (5.487-9.461) | <0.001 | 8.867 (4.468-17.598) | <0.001 |
| **Vomiting and nausea** | 3 (0-13) | 5 (0-38) |  |  |  |  |
| 0-0 |  |  | 1.000 |  | 1.000 |  |
| 1-3 |  |  | 0.679 (0.374-1.232) | 0.203 | 0.623 (0.323-1.200) | 0.157 |
| 4-24 |  |  | 0.940 (0.561-1.578) | 0.816 | 0.794 (0.445-1.418) | 0.436 |
| 25-100 |  |  | 1.891 (1.138-3.143) | 0.014 | 1.492 (0.843-2.642) | 0.169 |
| **Influence on**  **daily life** | 15 (2-49) | 50 (19-68) |  |  |  |  |
| 0-8 |  |  | 1.000 |  | 1.000 |  |
| 9-27 |  |  | 5.088 (2.683-9.650) | <0.001 | 4.933 (2.484-9.797) | <0.001 |
| 28-59 |  |  | 6.596 (3.445-12.628) | <0.001 | 6.226 (3.088-12.555) | <0.001 |
| 60-100 |  |  | 14.071 (7.313-7.074) | <0.001 | 11.297 (5.607-22.762) | <0.001 |
| **Psychological**  **well-being** | 15 (5-30) | 25 (10-56) |  |  |  |  |
| 0-4 |  |  | 1.000 |  | 1.000 |  |
| 5-15 |  |  | 0.862 (0.572-1.301) | 0.480 | 0.821 (0.534-1.261) | 0.367 |
| 16-30 |  |  | 1.452 (0.998-2.115) | 0.051 | 1.369 (0.926-2.025) | 0.115 |
| 31-100 |  |  | 2.721 (1.910-3.877) | <0.001 | 2.317 (1.589-3.376) | <0.001 |

OR = Odds Ratio. CI = Confidence Interval. VAS-IBS = visual analog scale for irritable bowel syndrome. Severity of symptoms from 0–100 mm, presented as median and interquartile range and categorized into quartiles [7,8]. Logistic regression adjusted for sex, smoking, drinking frequency, and drinking amount. Values are presented as OR and 95% CI. P-value < 0.05 was considered statistically significant.

**a**

|  | **β** | **95% CI** | **P-value** |
| --- | --- | --- | --- |
| **Constipation** |  |  |  |
| Sleeping onset difficulties 1-2 per week | 11.550 | 2.742-20.358 | 0.010 |
| **Bloating and flatulence** |  |  |  |
| Sleeping onset difficulties <1 per week | 10.505 | 2.962-18.048 | 0.006 |
| **Vomiting and nausea** |  |  |  |
| Wake-up frequency almost every night | 16.591 | 6.587-26.595 | 0.001 |
| **Psychological well-being** |  |  |  |
| Chronic stress 1 year | 11.331 | 9.486-13.175 | <0.001 |
| Chronic stress 5 years | 3.176 | 1.315-5.037 | 0.001 |
| Good sleeping quality | 3.103 | 0.927-5.278 | 0.005 |
| Average sleeping quality | 5.620 | 2.896-8.343 | <0.001 |
| Bad sleeping quality | 12.407 | 8.127-16.686 | <0.001 |
| Very bad sleeping quality | 15.737 | 6.663-24.811 | 0.001 |
| Sleeping onset difficulties 3-6/week | 6.553 | 2.233-10.872 | 0.003 |
| Sleeping onset difficulties almost every night | 10.179 | 5.211-15.148 | <0.001 |

β = beta-value. CI = confidence interval. GI = gastrointestinal. Symptoms the past 2 weeks were assessed by the visual analog scale for irritable bowel syndrome (VAS-IBS) where 0 mm means no symptoms and 100 mm means maximal symptoms [7,8]. Generalized linear model in a full model with all variables of stress and sleeping habits, adjusted for age, sex, occupation, and smoking. Values are presented as β and 95% CI. P-values ≤ 0.01 was considered as statistically significant.
